# A Historical Review of Changes in Nutrition Standards of USDA Child Meal Programs Relative to Research Findings on the Nutritional Adequacy of Program Meals and the Diet and Nutritional Health of Participants: Implications for Future Research and the Summer Food Service Program

**DOI:** 10.3390/nu7125523

**Published:** 2015-12-04

**Authors:** Laura C. Hopkins, Carolyn Gunther

**Affiliations:** 1 Department of Human Sciences–Human Nutrition, College of Education and Human Ecology, The Ohio State University, 1787 Neil Avenue, RM 262B, Columbus, OH 43210, USA; hopkins.774@osu.edu; 2 Department of Human Sciences–Human Nutrition, College of Education and Human Ecology, The Ohio State University, 1787 Neil Avenue, RM 313, Columbus, OH 43210, USA

**Keywords:** USDA child meal programs, childhood obesity, dietary intake, meal quality

## Abstract

Background: The USDA child meal programs (CMPs) (National School Lunch Program (NSLP), School Breakfast Program (SBP), and Summer Food Service Program (SFSP) were established in 1946 (NSLP) and 1975 (SBP and SFSP) to improve the diet and nutritional health of US children. There is concern that participation in these programs may in fact be a contributor to the current childhood obesity epidemic. Objective: The purpose of this study was to determine if the CMPs are meeting their intended goal by reviewing the historical changes to nutrition standards of the CMPs in correspondence with the literature that examines the nutritional adequacy of meals served as part of these programs, as well as the dietary intakes and nutritional status of participants. Methods: Public Law and the Federal Register were reviewed and websites and online databases were systematically searched. Results: NSLP and SBP first underwent updates to the nutrition standards in 1994 and subsequently 2010, whereas SFSP last underwent modifications in 2000. The majority of data, all collected prior to 2010, demonstrate that meals served as part of the NSLP and SBP are not meeting nutrition standards. In addition, the dietary intakes of NSLP and SBP participants are high in calories, fat, saturated fat, and sodium, and low in fiber. Studies examining the weight status and other nutrition-related health outcomes of NSLP and SBP participants have produced mixed results. In contrast, no studies published in the peer-reviewed literature have been conducted examining the nutritional adequacy of SFSP meals or the dietary intakes or nutritional health of SFSP participants. There are public reports available on the nutritionally adequacy of SFSP meals, however, they are severely outdated (1988 and 2003). Due to this dearth of information, a case study on a sample SFSP menu from summer 2015 was conducted; results showed that the meals are high in carbohydrate and protein content and insufficient in vegetable servings. Conclusions: There is critical need for policy change that would enable updates to the SFSP nutrition standards to match those of the NSLP and SBP. In addition, strategies are needed to facilitate development of CMP menus that meet current nutrition standards. Finally, rigorously designed studies are needed to understand the direct impact of CMP participation on child diet and health, particularly the SFSP for which there is limited published data.

## 1. Introduction

There has been a major shift in the nutritional status of our nation’s children over the past century from emaciation to obesity. The early 1900s were plagued with underfed children due to the consequences of two World Wars and the Great Depression. Feeding programs date back to the late 1700s, however, formal federal intervention did not occur until the 1930s during the Great Depression when there was a surplus of food produced from farms. At that time (1935), Public Law 320 was passed by Congress that enabled the government to purchase excess foods from the market and channel them elsewhere [1,2]. School lunch programs became an optimal target for this commodity surplus, which led to their subsequent expansion. However, this lasted for several years, at which time World War II occurred and surplus foods shifted to being shipped overseas, diminishing the supply for school lunch programs. Also, due to the negative effect of the war on the US economy, the government was unable to appropriate the necessary funds to continue to grow school lunch programs [2].

Fortunately, despite the lack of resources, Congress recognized the need for such child feeding programs, making the halt to the expansion of school lunch programs only temporary. In 1946, Congress signed into law the National School Lunch Act (NSLA), which first established the National School Lunch Program (NSLP) [2,3]. In the decades that followed, similar programs were formalized by the government. In 1966, the Child Nutrition Act (CNA) was signed into law, of which Section 4 established a pilot of the School Breakfast Program (SBP) [4]. Section 13 of the NSLA was amended in 1968 to pilot the Summer Food Service Program (SFSP) [5]. In 1975, SBP and SFSP were made permanent by an amendment to the CNA [6].

The NSLP, SBP, and SFSP are intended to address issues of malnutrition and food insecurity from a social ecological theory approach [7]. To this end, these child meal programs (CMPs) are mandated, overseen, and funded at the institutional level by the USDA. The policies, including policies of participation, implementation, and nutritional quality of meals of these national programs depend on community-level agencies such as school districts and food service operations. Interpersonally, provisions of meals to children at school and during the summertime inherently affect the food security status of households as a whole. Finally, the health and nutritional status of low-income children are dependent upon receipt of meals from these programs.

While CMPs were introduced with the goal of improving the diet and nutritional health of US children, there is concern that participation in these programs, due in part to the inadequate nutrition standards of the meals, may in fact be a contributor to the current childhood obesity epidemic—which affects 17% of US youth [8]—and poor dietary trends among school aged children [9,10,11]. With 11.2 million, 21.5 million, and 3 million US children participating in the NSLP, SBP, and SFSP, respectively, a major financial investment by the federal government, and considering the current childhood obesity epidemic, it is essential to ascertain that these federal programs, which are aimed at making children healthier, are in fact meeting their intended goal and not worsening children’s nutritional status [12]. The purpose of this study was to determine if the CMPs are achieving their mission by reviewing the historical changes to nutrition standards of the CMPs in reference to the literature that examines the nutritional adequacy of meals served are part of these programs, as well as the dietary intakes and nutritional status of participants.

## 2. Experimental Section

To assess the historical progress of the USDA CMPs (Enactments and Nutrition Standards Revisions), Public Law and the Federal Register were reviewed. The Library of Congress was referenced to obtain Public Law records for the NSLA of 1946 and the CNA of 1966 [3,4]. For the purposes of this review, two amendments to Public Law and four issues of the Federal Register were referenced to track changes to rules and regulations regarding these Acts [5,6,13,14,15,16].

For the review of nutritional adequacy of CMP meals and dietary intakes and nutritional outcomes of CMP participants, an extensive search of online databases was conducted to gather published articles. PubMed [17], ScienceDirect [18], EBSCOhost [19], and GoogleScholar [20] were searched using key words, in various combinations, including, but not limited to: National School Lunch Program, School Breakfast Program, Summer Food Service Program, USDA, participation, school meals, quality, overweight, obese, dietary intake, and children. No parameters were set for years searched. Approximately 816 unique articles matched these search criteria. The titles and abstracts of all of these articles were briefly scanned and filtered for research relevance and quality as outlined below (1–4), which resulted in 42 articles. Each of these articles were read in entirety. References were reviewed for additional publications. Twenty-three of the articles were eliminated due to: (1) inability to decipher independent effects of SBP or NSLP participation (*i.e.*, other food assistance programs were jointly assessed with SBP and NSLP); (2) studies pertaining only to the feasibility of universal-free SBP; (3) limited scope of dietary outcomes (*i.e.*, only assessed ready-to-eat cereal consumption); and (4) untrustworthiness of data presented (limited disclosure of statistical results). This resulted in a total of 19 articles that were included in this review. An overview of these studies is provided in Table 1. The search focus of the current study was placed on the peer-reviewed literature. In the case of missing information on a particular CMP within a given timeframe, a search of USDA websites was conducted.

For the final portion of this study, due to the finding that there is a dearth of information related to the SFSP, a case study was conducted to assess the nutritional adequacy of SFSP breakfasts and lunches. Using the Nutrition Data System for Research (NDSR) [21], a nutrient composition analysis was conducted on a one-week sample menu of breakfasts and lunches from July 2014 offered by an SFSP sponsor located in Columbus, Ohio that reaches over 340 open SFSP feeding sites. The nutritional composition reports from the analysis were compared to current NSLP and SBP nutrition standards, since current SFSP nutrition standards are outdated.

## 3. Results

### 3.1. History of Changes to the Nutrition Standards of USDA the National School Lunch Program (NSLP), School Breakfast Program (SBP), and Summer Food Service Program (SFSP)

**Figure 1:**
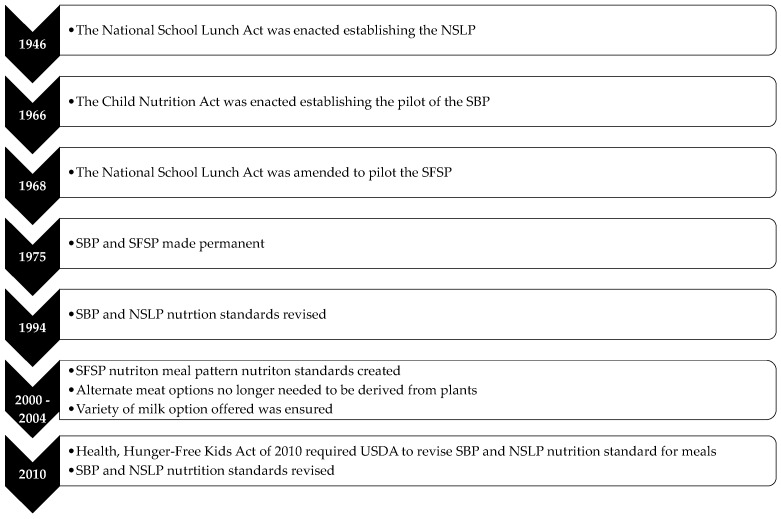
Historical Overview of the Policy and Programmatic Events that Occurred in the USDA National School Lunch Program (NSLP), School Breakfast Program (SBP), and Summer Food Service Program (SFSP) by Year.

Since the establishment of the NSLP (1946) and SBP (1975), the meal requirements and nutritional standards of these CMPs have changed significantly. Figure 1 provides an overview of the changes. Section 9 of the NSLA addressed the nutritional standards of the NSLP meals, stating that the lunches should meet minimum nutrition standards set by the Secretary on the basis of current research [3]. Based on this policy, the first NSLP food-based meal pattern requirements were created (Table 2). Type A meal requirements aimed to meet one-third of children’s daily food requirements, whereas Types B and C meals were intended to be supplemental [2]. Section 4 of the CNA outlined the nutrition standards of the SBP, which mirrored the language in the NSLA [4]. The SBP was made permanent in 1975, and no changes were made to the SBP nutrition standards at that time. When the NSLA was amended in 1968 to pilot the SFSP, no nutritional requirements for the SFSP were specified [5].

The first changes to the nutrition standards of the NSLP and SBP meals were made in 1994 as a result of USDA’s launch of the School Meals Initiative for Healthy Children which required nutritional improvements to school meals based on the new dietary guidelines [22]. The proposed changes shifted meals from a traditional food-based meal pattern to an enhanced food-based meal pattern that included specific nutrition guidance. The new nutrition standards regulations for the NSLP included: (1) Averaged over the course of a one-week period, provision of lunches meeting: (a) one-third of the Recommended Dietary Allowance (RDA) for calories; (b) one-third of the RDA for key nutrients including protein, calcium, iron, Vitamin A, and Vitamin C; (c) ≤30% of calories from fat; and (d) ≤10% of calories from saturated fat; (2) Reduction in sodium and cholesterol, albeit without quantifiable targets; (3) Increase in dietary fiber; however, no quantifiable targets were established; and (4) Inclusion of fluid milk, an entrée, and at least one other item to qualify as a meal [23]. Similar standards were set for the SBP with a target of meeting 25% of RDAs for calories and key nutrients. Table 3 outlines the 1994 SBP and NSLP nutrition standards.

**Table 1:** Summary of Studies Published in the Peer-Reviewed Literature Assessing the Nutritional Adequacy of the USDA National School Lunch Program (NSLP) and School Breakfast Program (SBP) Meals and Dietary Intakes and Nutritional Outcomes of NSLP and SBP Participants in Correspondence with Historical Events (Enactments and Nutrition Standard Revisions) of the NSLP and SBP ^1^.

Study	Design	Setting	Participants	Purpose	Outcomes Assessed	Findings
**Enactment of the National School Lunch Act (1946) and Child Nutrition Act (1966, 1975)**						
Paige, 1972	Observational; Prospective	• 4 elementary schools • Baltimore City, MD	• *n* = 742 1st, 2nd and 6th graders	• To assess nutritional impact on biomarkers and anthropometrics of NSLP participants compared to non-participants	• Height • Weight • Hematocrit	• NSLP participants were not nutritionally better off compared to non-participants
Hanes, 1984	Observational; Cross-sectional	• Nationally representative sample (NESNP ^5^)	• *n* = 1089 second graders	• Assess differences in caloric and nutrient intakes of NSLP participants and non-participants • Assess whether differences are due to food quality or quantity	• Dietary intake (24-h dietary recall)	• NSLP participants *vs.* non-participants consumed greater calories and amounts of all nutrients except vitamin C and iron • SBP participants *vs.* non-participants consumed more calcium, phosphorus, protein, and magnesium, but less iron, vitamin A, and vitamin B6
Vermersch, 1984	Observational; Cross-sectional	• Nationally representative sample (NESNP ^5^)	• *n* = 6556	• Assess differences anthropometrics between NSLP and SBP participants and non-participants	• Height • Weight • Triceps skinfold	• NSLP participants’ weight-for-age and body fat were significantly greater compared to non-participants • Participant in the SBP may move students away from the extremes to normal for weight and fat (triceps skinfold) distributions
Wolfe, 1994 ^2^	Observational; Cross-sectional	• New York State, excluding New York City • Schools randomly chosen	• *n* = 1797 2nd and 5th graders	• Determine weight distribution of children in New York State • Determine factors associated with child fatness	• Height • Weight • Triceps skinfold • Midarm circumference	• Higher BMI-percentile and arm fat area (midarm circumference) were significantly higher among NSLP participants compared to non-participants
Burghardt, 1995 ^2^	Observational; Cross-sectional	• Nationally representative sample (SNDA ^4^ I)	• *n* = 524 schools • *n* = 3350 students grades 1–12 from 329 schools	• Summarize key findings of the SNDA ^4^ I study • Assess dietary intake of NSLP/SBP participants compared to non-participants	• Nutrient analysis of meals offered • Dietary intake (24-h dietary recall)	• 1% of schools reached the target of <30% calories from fat for lunch • One of 544 reached the target of <10% of calories from saturated fat for lunch • Average calories from fat in the SBP meals was 14% • Both breakfasts and lunches exceeded sodium target
Melnick, 1998 ^2^	Observational; Cross-sectional	• New York City Schools	• *n* = 1396 2nd and 5th graders	• Assess the prevalence of overweight and obesity • Assess the relationship between weight status and household characteristics, including participation in NSLP/SBP	• Height • Weight • Household characteristics	• No association between free- or reduced-cost school lunch participation and weight status
**1994 Revisions to NSLP and SBP Nutritional Standards ^9^**						
Gleason, 2003	Observational; Cross-sectional	• Nationally representative sample (CSFII ^6^)	• *n* = 1021 children 6–18 years old	• Assess the relationship between NSLP participation and dietary intake	• NSLP participation • Dietary intake (24-h recall)	• NSLP participants consumed significant more calories, total fat, saturated fat, cholesterol, and sodium compared to non-participants • NSLP participants consumed significantly more than non-participants for several vitamins and minerals as well as less added sugar and total carbohydrate
Jones, 2003	Observational; Cross-sectional	• Nationally representative sample (PSID ^8^)	• *n* = 722 children aged 5–12 years old	• Assess the relationship between NSLP and SBP participation and weight status while considering food security status	• Household food security • School meal participation • Height • Weight	• Food insecure girls who participate in NSLP or combined NSLP and SBP have reduced odds of being overweight
Hofferth, 2005	Observational; Cross-sectional	• Nationally representative sample (PSID CDA ^8^)	• *n* = 1268 children aged 6–12 years old	• Assess the relationship between NSLP and SBP participation and weight status	• School meal participation • Height • Weight	• No significant relationships demonstrated between school meals participation and BMI-percentile or weight status
Addison, 2006	Observational; Cross-sectional	• 2 Mississippi school districts—one suburban and one urban	• n/a	• Examine diet quality compared to nutritional standards	• Nutrient analysis of menus	• Calories, fat, protein, and sodium were served in excess of recommended amounts
Clark, 2009	Observational; Cross-sectional	• *n* = 287 public schools • Nationally representative sample (SNDA ^4^ III)	• *n* = 2314 1st–12th graders	• Summarize meal analysis and dietary analysis results from SNDA ^4^ III	• Dietary intake (24-h dietary recall)	• More than three-quarters of school-aged children consume excessive amounts of saturated fat and sodium • NSLP and SBP participants consume significantly more calories than non-participants • A significantly greater percent of participants consume excessive amounts of sodium
Crepinsek, 2009	Observational; Cross-sectional	• *n* = 398 schools (130 school districts) • Nationally representative sample (SNDA ^4^ III)	• n/a	• Assess the nutrient content of NSLP and SBP meals	• Nutrient analysis of meals offered and served	• NSLP lunches were high in fat and saturated fat compared to standards • Both NSLP and SBP meals were high in sodium and low in fiber compared to standards
Gleason, 2009	Observational; Cross-sectional	• Nationally representative sample (SNDA ^4^ III)	• *n* = 2228 1st–12th graders	• Assess the relationship between school meal participation and weight status	• School meal participation • Height • Weight	• There was a significant, inverse relationship between SBP participation and BMI z score
Baxter, 2010 ^3^	Observational; Cross-sectional	• 17 schools in South Carolina	• *n* = 1780; 4th graders	• Investigate a potential relationship between BMI and participation in the NSLP and SBP	• Height • Weight • Observed meal intake	• No significant relationship between BMI-percentile and participation in NSLP and SBP • Average BMI-percentile was larger for children who ate breakfast in the classroom *vs.* the cafeteria
Paxton-Aiken, 2012 a ^3^	Observational; Cross-sectional (compilation of 4 studies)	• 6 to 11 elementary schools • Augusta, Georgia	• *n* = 1535 (meal participation data) 4th graders • *n* = 342 (dietary intake data) 4th graders	• Assess the relationship between NSLP and SBP participation and weight status • Assess the relationship between BSP and NSLP participation and dietary intake	• School meal participation (reported by researchers) • Height • Weight • Dietary intake (meal observation)	• Significant, positive relationship between SBP and NSLP participation and caloric intake was seen
Paxton, 2012 b ^3^	Observational; Cross-sectional (compilation of 4 studies)	• 13 elementary schools • Augusta, Georgia	• *n* = 1496 4th graders	• Assess the relationship between NSLP and SBP participation and weight status	• School meal participation (reported by parents) • Height • Weight	• Significant, positive relationship between SBP and BMI-percentile was seen • Significant, negative relationship between NSLP participation and BMI-percentile was seen
Hanson, 2013 ^3^	Observational; Cross-sectional	• Nationally Representative Sample (NHANES ^7^ 2003–2008)	• *n* = 2376 children aged 6–17 years old	• Assess the relationship between SBP and NSLP participation and weekday caloric intake and diet quality	• School meal participation • Dietary intake (24-h dietary recall) • Healthy Eating Index scores	• Total vegetable and milk component scores were significantly higher for participants • Whole grains, saturated fat, and sodium component scores were significantly lower for participants
**2010 Revisions to NSLP and SBP Nutritional Standards ^10^**						
Cohen, 2014	Observational; Prospective	• Urban district of Massachusetts	• *n* = 1030 elementary- and middle school-aged children	• Compare food selection, consumption, and waste prior to and after the implementation of new NSLP and SBP meal standards	• Tray plate waste methodologies to determine food selected, consumed, and wasted	• Fruit selection increased and milk selection decreased significantly • Entrée and vegetable consumption increased and milk consumption decreased significantly
Ohri, 2014	Observational; Cross-sectional	• New Jersey • Schools randomly chosen	• *n* = 1220 parents of school-going children	• Assess parental perceptions of NSLP lunches • To determine relationships between parents’ perceptions of NSLP lunches and their children’s consumption of NSLP lunches	• NSLP participation rate • Parental perceptions of the healthiness of NSLP lunches	• The students’ odds of consuming NSLP lunches was significantly lower if parents’ perceived the meals to be somewhat unhealthy or very unhealthy compared to parents who perceived the meals to be very healthy

^1^ Quality of meals and dietary intake and nutritional quality of SFSP participants are not included in this review due to a complete lack of peer-reviewed literature; ^2^ Dates of data collection for study were prior to 1994 meal requirement changes; ^3^ Dates of data collection for study were prior to 2010 meal requirement changes; ^4^ SNDA = School Nutrition Dietary Assessment; ^5^ NESNP = National Evaluation of School Nutrition Programs; ^6^ CSFII = Continuing Survey of Food Intakes by Individuals; ^7^ NHANES = National Health and Nutrition Examination Survey; ^8^ PSID = Panel Study of Income Dynamics; CDS = Child Development Supplement; ^9^ 1994 Changes: (1) Averaged over the course of a one-week period, provision of lunches meeting: (a) one-third of the Recommended Dietary Allowance (RDA) for calories; (b) one-third of the RDA for key nutrients including protein, calcium, iron, vitamin A, and vitamin C; (c) ≤30% of calories from fat; and (d) ≤10% of calories from saturated fat; (2) Reduction in sodium and cholesterol; however, quantifiable targets were not established; (3) Increase in dietary fiber; however, no quantifiable targets were established; and (4) inclusion of fluid milk, an entrée, and at least one other item to qualify as a meal [23]; ^10^ 2010 Changes: (1) Requirement of fruits to be a separate meal component; (2) Requirement of vegetables to be a separate meal component; (3) Offer vegetables daily at lunch with subgroups (dark green, red/orange, legumes, starchy, other); (4) Limit starchy vegetables; (5) Requirement of grains to be whole grains half of the time and progressing to an all of the time after two years; (6) Serve fat-free (flavored and unflavored) and low-fat (unflavored) milk only; (7) Meet specific calorie ranges for age and grade groups; (8) Reduce sodium incrementally over a 10-year period; (9) Eliminate trans-fat; and (10) Narrow the ranges of age and grade groups [16].

**Table 2:** The 1946 USDA National School Lunch Program (NSLP) Food-Based Meal Patterns per Day.

Food Group	Type A Serving Size	Type B Serving Size	Type C Serving Size
Milk, whole	½ pint	½ pint	½ pint
Protein-rich food:			
Fresh or processed meat, poultry, cheese, cooked or canned fish	2 oz.	1 oz.	
Dry peas or beans, soy beans (cooked)	½ cup	¼ cup	
Peanut Butter	4 Tbsp	2 Tbsp	
Eggs	1	½	
Raw, cooked, or canned vegetables or fruits	¾ cup	½ cup	
Bread, muffins, or hot bread ^1^	1 portion	1 portion	
Butter or fortified margarine	2 tsp	1 tsp	
Source: replicated from Gunderson, 1971 [2]			

^1^ Whole grain cereal or enriched flour.

**Table 3:** An Overview of the 1994 School Breakfast Program (SBP) and National School Lunch Program (NSLP) Weekly Nutrition Standards.

	Breakfast	Lunch	
Food Group	K-12	K-3	4–12
**Fruit (cups)**	2.5	2.5 ^3^	3.75 ^3^
**Vegetable (cups)**	0	0	0
**Grain/Bread (oz. equivalent) ^1^**	0–10 ^2^	8 ^1,4^	8 ^1,4^
**Meat/Vegetable Protein Product**	0–10 ^2^	7.5	10
**Milk (cups)**	5	5	5
**SBP Nutrient Requirements:** Meet 25% of RDAs ^5^ for key nutrients ^6^; <30% calories from fat; <10% calories from saturated fat			
**NSLP Nutrient Requirements:** Meet 33.3% of RDAs ^5^ for key nutrients ^6^; <30% calories from fat; <10% calories from saturated fat			
Source (replicated from): USDA Food and Nutrition Service [16]			

^1^ Whole grain or enriched; ^2^ SBP require two grains, two meats, or one of each; ^3^ Plus an extra half cup at some point throughout the week; ^4^ Minimum of one per day; ^5^ Recommended Dietary Allowance; ^6^ Protein, calcium, iron, Vitamin A, and Vitamin C.

From 2000 to 2004, minor changes to the NSLP and SBP nutrition standards were made, though they did not involve altering nutrient content of the meals (e.g., establishing definitions for: vegetable protein products; blended beef, pork, poultry, and seafood products; fluid milk products) [13,14,15]. In 2000, food-based meal standards were created for the SFSP, which are reflected in Table 4.

**Table 4:** USDA Summer Food Service Program (SFSP) Breakfast and Lunch Requirements per Day.

Number of Servings per Food Group	Serving Size	Qualifying Foods
**Breakfast ^1^**		
1 Milk	1 cup	Fluid milk
1 Fruit/Vegetable	½ cup	Juice ^2^, fruit or vegetable
1 Grain ^3^	1 slice	Bread
	1 serving	Cornbread, biscuit, roll, or muffin
	¾ cup	Cold, dry cereal
	½ cup	Hot, cooked cereal
	½ cup	Pasta, noodles, or other grains
**Lunch or Supper ^1^**		
1 Milk	1 cup	Fluid milk
2 Fruit/Vegetable	¾ cup	Juice ^2^, fruit or vegetable
1 Grain ^3^	1 slice	Bread
	1 serving	Cornbread, biscuit, roll, or muffin
	½ cup	Hot, cooked cereal
	½ cup	Pasta, noodles, or other grains
1 Meat/Meat Alternate	2 oz.	Lean meat, poultry, or fish ^4^
	2 oz.	Alternate protein product
	2 oz.	Cheese
	1 Large	Egg
	½ cup	Cooked, dry beans or peas
	4 Tbsp	Peanut or other nut/seed butter
	1 oz.	Nuts or seeds ^5^
	8 oz.	Yogurt ^6^
**Snacks ^1^**		
1 Milk	¾ cup	Fluid milk
2 Fruit/Vegetable	¾ cup	Juice ^7^, fruit or vegetable
1 Grain ^3^	1 slice	Bread
	1 serving	Cornbread, biscuit, roll, or muffin
	¾ cup	Cold, dry cereal
	½ cup	Hot, cooked cereal
	½ cup	Pasta, noodles, or other grains
1 Meat/Meat Alternate	1 oz.	Lean meat, poultry, or fish ^4^
	1 oz.	Alternate protein product
	1 oz.	Cheese
	½ Large	Egg
	¼ cup	Cooked, dry beans or peas
	2 Tbsp	Peanut or other nut/seed butter
	1 oz.	Nuts or seeds
	4 oz.	Yogurt ^6^
Source (replicated from): USDA Food and Nutrition Service [24]		

^1^ USDA SFSP sites can only be reimbursed for 2 types of meals, *i.e.*, Breakfast and Lunch or Breakfast and 1 Snack; ^2^ Full strength fruit or vegetable juice; ^3^ Whole grain or enriched meal/flour; Cereal must be whole grain, enriched, or fortified; ^4^ Edible portion of cooked lean meat, poultry, or fish; ^5^ Nuts and seeds may meet only one-half of the total meat/meat alternate serving and must be combined with another meat/meat alternate to fulfill the lunch or supper requirement; ^6^ Plan or flavored; Unsweetened or sweetened; ^7^ Full strength fruit or vegetable juice; Cannot be served when milk is the only other snack component.

The Healthy, Hunger-Free Kids Act of 2010 required the USDA to update NSLP and SBP nutrition standards [25]. Significant changes were made and included the following [16]: requiring fruits and vegetables to each be a separate meal component; offering vegetables daily (at lunch) with subgroups; limiting starchy vegetable; requiring grains to be whole grains “half of the time” and progressing to “all of the time” after two years; serving fat-free (flavored and unflavored) and low-fat (unflavored) milk only; specifying calorie ranges for age and grade groups; reducing sodium reduction over a 10-year period; eliminating trans-fat; and narrowing age and grade groups [16]. In addition to these outlined changes regarding the nutritional standards of meals, an initiative to monitoring the changes was implemented as well, including a three-year review of breakfasts and lunches [16]. Table 5 provides an overview of the most recent nutritional standards for the NSLP and SBP.

**Table 5:** Current School Breakfast Program (SBP) and National School Lunch Program (NSLP) Weekly Nutrition Standards.

	Breakfast; Amount per Week (Min. per Day)			Lunch; Amount per Week (Min. per Day)		
**Food Group**	**K-5**	**6–8**	**9–12**	**K-5**	**6–8**	**9–12**
**Fruit (cups) ^1,3^**	5 (1)	5 (1)	5 (1)	2½ (½)	2½ (½)	5 (1)
**Vegetable (cups) ^2,3^**	0	0	0	3¾ (¾)	3¾ (¾)	5 (1)
***Dark Green***	0	0	0	½	½	½
***Red/Orange***	0	0	0	¾	¾	1¼
***Beans/Peas (Legumes)***	0	0	0	½	½	½
***Starchy***	0	0	0	½	½	½
***Other***	0	0	0	½	½	¾
**Additional Vegetables to Reach Total ^4^**	0	0	0	1	1	1½
**Grain/Bread (oz. equivalent) ^5^**	7–10 (1)	8–10 (1)	9–10 (1)	8–9 (1)	8–10 (1)	10–12 (2)
**Meat/Vegetable Protein Product**	0 ^6^	0 ^6^	0 ^6^	8–10 (1)	9–10 (1)	10–12 (2)
**Milk (cups) ^7^**	5 (1)	5 (1)	5 (1)	5 (1)	5 (1)	5 (1)
	**Other Specifications**					
**Min-Max Calories (kcal)**	350–500 ^8^	400–500 ^8^	450–600 ^8^	550–650 ^8^	600–700 ^8^	750–850 ^8^
**Saturated fat (% of total calories)**	<10	<10	<10	<10	<10	<10
**Sodium (mg) ^9^**						
*2014–2015 Target*	≤540 ^9^	≤600 ^9^	≤640 ^9^	≤1230 ^9^	≤1360 ^9^	≤1420 ^9^
*2017–2018 Target*	≤485 ^9^	≤535 ^9^	≤570 ^9^	≤935 ^9^	≤1035 ^9^	≤1080 ^9^
*2022–2023 Target*	≤430 ^9^	≤470 ^9^	≤500 ^9^	≤640 ^9^	≤710 ^9^	≤740 ^9^
**Trans fat**	0 gram of trans fat per serving must be indicated on nutrition label or by manufacturer for all food and beverage products					
**Nutrients**	SBP: meet 25% of RDAs ^10^ for nutrients; NSLP: meet 33.3% of RDAs ^10^ for nutrients					
Source (replicated from): USDA Food and Nutrition Service [16]						

^1^ ¼ cup of dried fruit is equivalent to ½ cup of fruit; ^2^ 1 cup of leafy greens is equivalent to ½ cup of vegetables; ^3^ Juice cannot fulfill more than half of the fruit or vegetable requirement; ^4^ Can consist of any of the vegetable subgroups; ^5^ Must be whole grains; ^6^ 1 oz. equivalent of meat/meant alternate may be substituted for 1 oz. equivalent of grains after minimum daily grain requirement is met; ^7^ Must be low-fat (unflavored) or fat-free (unflavored or flavored); ^8^ Values provided are for the average daily amount per week and must be within range or less than indicated; ^9^ Sodium targets become gradually more stringent over time; ^10^ Recommended Dietary Allowance.

### 3.2. Nutritional Adequacy of CMP Meals as Well as Dietary Intakes and Nutritional Outcomes of USDA CMP Participants within the Specific Timeframes that Coincide with Changes to Nutrition Standards of USDA CMPs

#### 3.2.1. Enactment of the NSLA (1946) and CNA (1966)

After the enactments of the NSLA and CNA, several independent researchers began investigating the potential biological effects of the universal school feeding programs. One study assessed first, second, and sixth graders in elementary schools in Baltimore City, MD over the course of a year to determine potential nutritional effects of NSLP participation by assessing height, weight, and hematocrit, an indicator of iron status that is commonly used to assess overall nutritional status [26]. Sixty percent of NSLP participants who started the school year with a low hematocrit level did not significantly improve their hematocrit level over the course of the school year [27]. Additionally, 10% of NSLP participants demonstrated a decline in hematocrit from normal to low throughout the course of the school year [27]. Thus, it was concluded that NSLP participants were not better off nutritionally compared to non-participants.

Within the indicated timeframe, a few studies examined NSLP and SBP participation and associations with weight status. Anthropometric data were assessed for participants in the National Evaluation of School Nutrition Programs (NESNP) study to determine if NSLP and SBP participation were associated with height-for-age, weight-for-age, and triceps skinfold (indicator of body fat). Results indicated that NSLP participants’ weight-for-age and body fat were significantly greater compared to non-participants (both *p* < 0.01) [28]. With regards to the SBP, it was demonstrated that frequent and long-term SBP participation may move students away from the extremes to mid-distributions for weight and fat body (*p* < 0.05) [28]. In a study assessing students from New York State, BMI-percentile and arm fat area were significantly greater for NSLP participants compared to non-participants (*p* < 0.05; *p* < 0.05) [29]. Contrary to these findings, Melnick *et al.* found no association between NSLP participation and overweight status among second and fifth graders in New York City [30].

Nutrition analyses of NSLP and SBP meals served and dietary intakes of children were also investigated within the given period of time. In the NESNP study, the impacts of federally funded nutrition programs were assessed [31]. According to the findings, NSLP participants consumed greater calories and amounts of all nutrients, except Vitamin C and iron [32]. The researchers attributed the greater consumption of nutrients to quality as opposed to quantity of foods served in the NSLP [31]. Additionally, SBP participants consumed more calcium, phosphorus, protein, and magnesium [31]. However, SBP participants consumed less iron, Vitamin A, and Vitamin B6 compared to non-participants [31].

Results from the first School Nutrition Dietary Assessment (SNDA) study indicated that the average percentage of calories from fat in NSLP lunches was 38% and only 1% of schools provided lunches that reached the target of <30% calories from fat [23]. The SBP meals were closer to the target with an average 31% of calories from fat [23]. Only one of 544 schools provided lunches that reached the target of <10% of calories from saturated fat, and the average calories from fat in the SBP meals were 14% [23]. The average sodium content of NSLP lunches was approximately twice the target (800 mg) at 1479 mg, and slightly over the target (600 mg) for SBP at 673 mg [23]. In addition to content analyses of the meals offered and served, dietary intakes of participants compared to non-participants were compared. Average dietary intakes of protein, Vitamin C, calcium, and most other nutrients for NSLP participants were above one-third of the RDAs compared to students who ate non-NSLP lunches or lunches brought from home [23]. For SBP participants, intakes of all nutrients were above the recommended one-fourth of the RDA, except for zinc [23]. However, female NSLP participants between the ages of 11 and 14 consumed less than one-third of the RDA for iron, magnesium, zinc, Vitamin A, and Vitamin B-6 [23]. More than the targets of fat, saturated fat, and sodium were consumed, on average, at lunch by NSLP participants [23]. For SBP participants, calorie and fat intake was within recommendations, however, saturated fat intake far exceeded recommendations [23].

In contrast to the NSLP and SBP, no studies published in the peer-reviewed literature examining the nutritional adequacy of SFSP meals or the diet and health of SFSP participants within the given timeframe were identified. A 1988 USDA ERS report utilizing a national dataset demonstrated that the vast majority of SFSP lunches contained the required meal components, though nutritional adequacy was not evaluated [32]. Plate waste at lunch was also measured as part of this study. Results demonstrated that food for four meal components (milk, fruits/vegetables, meat, and bread) was wasted from 20% to 36%, with milk and fruits/vegetables being wasted most often.

#### 3.2.2. 1994 Changes to the NSLP and SBP Nutritional Standards

Eleven studies were included in this review that examined school meals and the nutritional outcomes of school meal participants after the new, revised nutritional standards were implemented in 1994. Two studies examined the nutritional content of the meals served, three studies examined the actual dietary intake of the children, and the remaining studies examined weight status.

Both regional and national samples of schools were used when assessing the nutritional content of the school meals. A nutritional analysis of menus in one urban and one rural school district in Mississippi was conducted and compared to SBP and NSLP nutritional standards. Calories, fat, protein, and sodium all surpassed recommended amounts. For both grade groups, K-6 and 7–12, in both the urban and rural districts, calories (*p* = 0.008; *p* = 0.003; *p* = 0.022; *p* = 0.038), fat (*p* = 0.009; *p* = 0.035; *p* = 0.009; *p* = 0.035), and protein (*p* = 0.004; *p* < 0.001; *p* = 0.001; *p* = 0.0001) were significantly greater than SBP and NSLP guidelines [33]. A more nationally representative sample was assessed using data from the SNDA III. Crepinsek *et al*. compared the nutritional content of meals offered and meals actually served. Approximately 23% and slightly less than half of surveyed schools actually served breakfasts and lunches, respectively, that met nutritional standards for calories [34]. Twenty percent and less than 33% of schools served NSLP meals that met the standards for fat and saturated fat, while approximately 80% and 70% of schools served SBP meals that met the standards for fat and saturated fat [34]. The mean sodium content of NSLP lunches was 1442 mg, which is slightly less than twice the recommended amount, with no schools meeting the requirement [34]. With regards to breakfast, approximately half of the schools met the sodium recommendation [34]. Less than 8% of schools provided lunches that met the recommendation for fiber and no schools provided breakfast that met the recommendation for fiber [34].

Three different national data sets were utilized in the studies examining the dietary intakes of school aged children, including both CMP (NSLP and SBP) participants and non-participants. Gleason *et al.* used the 1994–1996 Continuing Survey of Food Intakes by Individuals (CSFII), the oldest of these three data sets. From this nationally representative sample of 6–18 year olds, it was found that NSLP participants compared to non-participations consumed significantly more calories (*p* < 0.01), total fat (*p* < 0.01), saturated fat (*p* < 0.01), protein (*p* < 0.01), thiamin (*p* < 0.05), riboflavin (*p* < 0.01), Vitamin B6 (*p* < 0.01), Vitamin B12 (*p* < 0.01), calcium (*p* < 0.01), phosphorus (*p* < 0.01), magnesium (*p* < 0.01), zinc (*p* < 0.01), cholesterol (*p* < 0.01), and sodium (*p* < 0.01) and significantly less added sugars (*p* < 0.01) and total carbohydrates (*p* < 0.01) at lunch, as well as over an entire 24-h period [35]. Similar results were seen using from SNDA III data set in a study conducted by Clark *et al.* When examining all school-aged children, 80% and 92% consumed excessive amounts of saturated fat and sodium, respectively [10]. SBP and NSLP participants consumed significantly more calories (*p* < 0.01), calcium (*p* < 0.05), and fiber (*p* < 0.01) and less magnesium (*p* < 0.01) and phosphorus (*p* < 0.001) compared to non-participants [10]. Additionally, high school-aged participants consumed significantly more sodium (*p* < 0.05) compared to non-participants [10].

The final study utilized a national data set and took a slightly different approach, but demonstrated similar trends with regards to sodium, fat, and caloric intake. Hanson *et al.* examined overall diet quality by applying the Healthy Eating Index (HEI) scoring system to data derived from the NHANES 2003–2008 survey. HEI is a validated measure of overall diet quality comprised of component scores for total fruit, whole fruit, total vegetables, greens and beans, whole grains, dairy, total protein foods, seafood and plant proteins, fatty acids, refined grains, sodium, and empty calories [36,37]. Higher scores and component scores indicate better diet quality. NSLP and SBP participants’ total vegetable (*p* < 0.01) and milk (*p* < 0.01) component scores were significantly higher, and whole grains (*p* < 0.01), saturated fat (*p* < 0.05), and sodium (*p* < 0.05) component scores were significantly lower than non-participants [38].

Findings regarding weight status were inconsistent across the reviewed studies. Investigators in South Carolina assessed the relationship between SBP and NSLP participation and BMI-percentile and found that there was no significant relationship between SBP, NSLP, or combined participation and BMI-percentile (*p* > 0.072). However, data indicated that children who ate breakfast in the classroom had significantly higher BMI-percentiles compared to children who ate breakfast in the cafeteria (*p* = 0.012) [39]. Two studies were conducted from the same data set of 4th graders in Augusta, Georgia. One study determined SBP and NSLP participation via researcher [40], while the other study determined SBP and NSLP participation through parental reporting [41]. In the first study, no significant relationships were identified between school meals participation and BMI-percentile [40]. However, significant, positive relationships were seen between SBP (*p* < 0.01), NSLP (*p* < 0.0001), and combined (*p* < 0.0001) participation and caloric intake [40]. Results from the parental reporting study found a positive, significant relationship between SBP participation and BMI-percentile (*p* = 0.05) and a negative, significant relationship between NSLP participation and BMI (*p* = 0.001) [41].

Results from studies that utilized national data sets were slightly more consistent, except with regard to SBP participation. In a study using the SNDA III data set, the relationships between SBP and NSLP participation and weight status were assessed. A negative, significant (*p* < 0.05) relationship was determined between SBP participation and BMI z score [42]. Positive, but insignificant, relationships were seen between NSLP participation and combined SBP and NSLP participation and BMI z score [41]. Similar null findings were seen by Hofferth *et al.* To this end, the PSID data set showed participation in NSLP was associated with a significantly higher BMI-percentile (*p* < 0.05) and being overweight (*p* < 0.10) in unadjusted models; however, when adjusted for demographical characteristics, the associations did not hold [43]. In another study using the PSID data set, Jones *et al.* examined the relationship between food assistance program participation weight status across groups of varying levels of household food security. Results from this study indicated that food insecure girls who participated in the NSLP or both the SBP and NSLP had 74% and 58% reduced odds of being overweight compared to food insecure non-participants [44].

Finally, no studies published in the peer-reviewed literature examining the nutritional adequacy of SFSP meals or the diet and health of SFSP participants within the given timeframe were identified. A 2003 USDA ERS report presenting 2001 data on a national sample demonstrated that SFSP breakfasts provided more than a quarter of the 1989 RDAs for protein, vitamin A, iron, calcium, and vitamin C, while SFSP lunches provided more than one third of the RDA for these nutrients [45]. On the other hand, SFSP breakfasts exceed the Dietary Guidelines recommendation for saturated fat and lunches exceeded the Dietary Guidelines for total fat and saturated fat. Plate waste data were also collected as part of this 2001 study. Results demonstrated that SFSP participants wasted approximately a third of the calories and nutrients served at both breakfast and lunch with the rate of waste being the highest for vegetables.

#### 3.2.3. 2010 Changes to the NSLP and SBP Nutritional Standards

Since the most recent revisions to the NSLP and SBP nutritional standards, only two studies have been published in the peer-reviewed literature. One study aimed to understand parents’ perceptions of the meals served at school and how these perceptions may relate to participation [46]. The odds of children consuming NSLP lunches were significantly lower if their parents’ perceived the meals to be somewhat unhealthy (*p* < 0.001) or very unhealthy (*p* = 0.057) compared to parents who perceived the meals to be very healthy [46]. The other study assessed the impact of the nutrition standards changes on food selection, consumption, and waste [47]. Results indicated that there was a significant increase in fruit selection (*p* < 0.0001) and significant decreases in milk selection and consumption (*p* < 0.0001; *p* < 0.001) after the new nutritional standards were implemented [47]. Entrée consumption increased significantly by 15.6% (*p* < 0.0001), and vegetable consumption increased significantly by 16.2% or 0.2 cups (*p* < 0.0001) [47]. Though not published in the peer-reviewed literature, it is also worth noting that results from the SNDA IV (2009–2010) were recently released, which showed that overall only 14% of surveyed schools met all of the nutrition standards for NSLP lunches, while the majority of schools met the standards for SBP breakfasts [47].

### 3.3. Case Study on a Sample SFSP Menu

Due to the finding that there are no data published in the peer-reviewed on the nutritional adequacy of SFSP meals, and given that the public reports on the SFSP are severely outdated (1988 [32] and 2003 [45]), a case study on the nutritional adequacy of SFSP meals from summer 2015 was conducted. The breakfast, lunch, and combined breakfast and lunch requirements for the K-5th, 6th–8th, and 9th–12th age groups for several key nutrients were compared to the mean (over a one-week period) amount of the nutrients in foods served, and the differences between the requirements and mean amount in foods served were calculated. The results for this analysis are presented in Table 6.

**Table 6:** Case Study on the Nutritional Adequacy of a Sample Summer Food Service Program (SFSP) Breakfast and Lunch Menu from July 2014 and Comparison to Requirements for National School Lunch Program (NSLP) and School Breakfast Program (SBP) ^1^.

Nutrient	Breakfast			Lunch			Combined Breakfast & Lunch		
	Requirement	Mean Served	Difference	Requirement	Mean Served	Difference	Total Daily Requirement	Total Mean Served	Difference
**Calories (kcal)**									
K–5th	350–500	411	+/−	550–650	745	+	900–1150	1156	+
6th–8th	400–550		+/−	600–700		+	1000–1250		+/−
9th–12th	450–600		−	750–800		−	1200–1400		−
**Carbohydrate (g/day)**	33	79	+	44	98	+	77	177	+
**Fiber (g/day)**									
K–5th	7	4	−	9	7	−	16	11	−
6th–8th	7–8		−	9–11		−	16–19		−
9th–12th	7–10		−	9–13		−	16–23		−
**Protein (g/day)**									
K–5th	3	10	+	4	26	+	7	36	+
6th–8th	4–9		+	5–12		+	9–21		+
9th–12th	5–13		+/−	7–18		+	12–31		+
**% Fat Calories**	<30%	15	−	<30%	34	+	<30%	24.5	−
**% Saturated Fat Calories**	<10%	7	−	<10%	11	+	<10%	9	−
**Sodium (mg/day) ^2^**									
K–5th	540	298	−	1230	1471	+	1770	1769	−
6th–8th	600		−	1360		+	1960		−
9th–12th	640		−	1420		+	2060		−
**Vitamin A (mcg/day)**									
K–5th	100	253	+	133	506	+	233	759	+
6th–8th	150		+	200		+	350		+
9th–12th	175–225		+	233–300		+	408–525		+
**Vitamin C (mg/day)**									
K–5th	7	8	+	9	14	+	16	22	+
6th–8th	12		−	15		−	27		−
9th–12th	17–19		−	17–22		−	34–41		−
**Vitamin D (mcg/day)**									
K–5th	4	3.5	−	5	4	−	9	7.5	−
6th–8th	4		−	5		−	9		−
9th–12th	4		−	5		−	9		−
**Calcium (mg/day)**									
K–5th	250	364	+	333	603	+	583	967	+
6th–8th	325		+	433		+	758		+
9th–12th	325		+	433		+	758		+
**Iron (mg/day)**									
K–5th	3	3.25	+	3	3.75	+	6	7	+
6th–8th	2		+	3		+	5		+
9th–12th	3–4		+/−	4–5		−	7–9		−
**Phosphorus (mg/day)**									
K–5th	125	303	+	167	603	+	292	906	+
6th–8th	313		−	417		+	730		+
9th–12th	313		−	417		+	730		+
**Zinc (mg/day)**									
K–5th	2	1.8	−	2	2.9	+	4	4.7	−
6th–8th	2		−	3		−	5		+
9th–12th	3		−	3–4		−	6–7		+

^1^ Nutrition Data System for Research (NDSR) was used for the nutrient composition analysis on the sample breakfast and lunch menus from July 2014 offered by an SFSP sponsor located in Columbus, Ohio that reaches over 340 open SFSP feeding sites. Nutritional composition reports compared to current NSLP and SBP nutrition standards, since current SFSP nutrition standards are outdated; ^2^ 2014–2015 sodium targets were used as comparison.

The percentage of calories from fat, percentage of calories from saturated fat, and sodium were below the required maximum amount for breakfast in all age groups, however, lunch provided more than the required maximum amount of these nutrients for all age groups. When combining the means for breakfast and lunch, percentage of calories from fat, percentage of calories from saturated fat, and sodium were below the required maximum amount for breakfast in all age groups. Individually and combined, the breakfasts and lunches provided excessive amounts of protein and carbohydrates and less than the required amount of fiber for all age groups. The findings indicated that, in general, meals served were within recommendations for most micronutrients. Worth noting, for older children, the meals served did not meet the recommendations for calories, fiber, Vitamin C, iron, and zinc. Finally, only two servings of vegetables were provided over the course of the entire week.

## 4. Discussion

There has been growing concern over the past several decades that participation in USDA school meals, due in part to inadequate nutritional standards of the meals, may be contributing to the current obesity epidemic and poor diet quality trends among children [9,10,11]. Even though nutritional requirements of CMPs have been modified based on some evidence and changes to the Dietary Guidelines for Americans, it is unclear as to whether the nutritional standards of these food assistance programs are strict enough to ensure adequate nutrition among school aged children. Given the high reliance of US children on these programs, a major financial investment by the federal government over the past nearly 60 years, and in light of the current childhood obesity epidemic, studies are needed to determine if these child food assistance programs—which are specifically designed to improve the nutrition and health of US children—are in fact accomplishing their goal. Without such studies, we can only hypothesize on the mission accomplishment. The aim of this study—which to our knowledge is the first of its kind—was to determine the programmatic progress and areas for improvement of the USDA CMPs by reviewing the historical changes to nutrition standards of the CMPs in reference to the literature that examines the nutritional adequacy of meals served are part of these programs, as well as the dietary intakes and nutritional status of CMP participants.

After these programs were established and prior to the first changes to the nutrition standards, several researchers began investigating the potential impacts of the implementation on child health. Only one study utilized biomarker technology to assess physiological impacts of the programs and the findings indicated no biological improvements and even some decreases in nutritional status among participants [27]. Three studies investigated weight status and indicators of body fat, which produced conflicting data. Vermeersch *et al.* and Wolfe *et al.* saw positive, significant associations with NSLP participation and weight status and body fat indices [28,29]. Interestingly, a potential protective effect of SBP participation on weight status was also demonstrated [28]. Contrary to these findings, Melnick *et al.* found no significant associations between NSLP participation and weight status [30]. However, in this study, the NSLP participation rate was 85% for the sample; thus, due to this high reported participation rate, it may have been difficult to detect an association [30].

None of the aforementioned reviewed studies that assessed biomarkers, weight status, or indicators of body fat of CMP participants conducted nutritional analyses of meals served or dietary intakes from the participants to investigate potential dietary reasons for their findings. However, during the same time period, two other studies investigated the nutritional content of meals served and dietary intake of school meal participants from larger, more nationally representative samples. Hanes *et al.* found that NSLP participants consumed greater calories and amounts of all nutrients except Vitamin C and iron and SBP participants consumed more calcium, phosphorus, protein, and magnesium, but less iron, Vitamin A, and Vitamin B6 compared to non-participants [31]. Similarly, Burghardt *et al.* found that female NSLP participants between the ages of 11 and 14 years old consumed less than one-third of the RDA for iron, magnesium, zinc, Vitamin A, and Vitamin B-6 [23]. Additionally, NSLP participants consumed more than the recommended amounts of fat, saturated fat, and sodium, and SBP participants consumed more than the recommended amount of saturated fat [23].

These nutrient analyses of meals consumed and dietary intakes may help explain the biological and physiological findings of Paige, Versmeersch, and Wolfe *et al*. [27,28,29]. The biomarker assessed in the sample of children in Baltimore, MD was hematocrit, an indicator of iron status. Both Hanes and Burghardt demonstrated that the meals and dietary intakes of NSLP and SBP participants contained iron below the recommended amounts [23,31]. Additionally, NSLP and SBP meals and participants’ intakes appeared to exceed recommendations for fat and saturated fat, which could explain the positive, significant associations between participation and weight status and body fat indices.

In 1994, new nutritional standards for the NSLP were established due in large part to the above-explained trends. These new requirements expanded the meal-pattern requirements to include specific nutrient-based requirements with a focus on meeting RDAs for key nutrients, reducing the fat, cholesterol, and sodium content, and increasing the fiber content of food served. After these changes were implemented, Crepinsek *et al.* examined a nationally representative sample of meals offered and served to SBP and NSLP participants. Almost one-quarter of breakfasts and almost half of lunches served did not meet the nutrition requirements with particularly low compliance for fiber, fat, saturated fat, and sodium [34]. School breakfasts tended to be more compliant than school lunches, which is likely due to the attainability of the requirements. These findings regarding the nutritional content of the meals served were supported by nutritional analyses of the dietary intakes of SBP and NSLP participants. Several researchers found significantly higher intakes of calories [10,33,35,37], leading to increased intake of total fat [35], saturated fat [35], cholesterol [35], and sodium [10,35], among participants compared to non-participants. However, it is worth noting that results from these studies also indicated significantly greater intake of several key vitamins and minerals among participants compared to non-participants, though this is likely due to the consumption of additional food [10,35]. These data demonstrate the need for strategies that would facilitate development of CMP menus that meet current nutrition standards.

In the papers reviewed for the current study, findings regarding the relationship between school meal participation and weight status varied. Several studies found positive but insignificant relationships between NSLP or combined SBP and NSLP participation and weight status [39,40,43]. Two studies were conducted from the same data set of 4th graders but determined participation in the SBP and NSLP by different means (researcher *vs.* parent report), which resulted in contradicting findings with regards to school meal participation and weight status. In one of the studies, no significant relationships were seen between school meals participation—SBP, NSLP, or combined—and BMI-percentile [40], while in the other study, a positive, significant relationship between SBP participation and BMI-percentile and an inverse, significant relationship between NSLP participation and BMI-percentile were demonstrated [41]. These contradicting results demonstrate the potentially deleterious effects of reporting bias with regards to self-reported data and observations from a potentially biased data collector—and the need for better designed studies eliminating potential biases.

The latter finding of a positive, significant relationship between SBP participation and BMI-percentile by Paxton-Aiken *et al.* also contradicts findings from two other studies. Consistent with Versmeersch *et al*. Gleason and colleagues found an inverse, significant relationship between SBP participation and BMI z score [42]. Breakfast skipping is prevalent in the US and elsewhere throughout the world. Several cross-sectional studies have demonstrated that adolescents who skip breakfast tend to have higher BMIs [29,48,49,50,51], however this is not a consistent finding [52,53,54]. If non-SBP participants are skipping breakfast, it is possible the participating in the SBP is providing a protective effect with regards to overweight and obesity. However, a cause and effect relationship between breakfast consumption and weight status cannot be determined from these studies.

Two other interesting findings regarding school meal participation and weight status were demonstrated in the current study. First, food insecure girls who participated in the NSLP or both the SBP and NSLP had a reduced odds of being overweight compared to food insecure non-participants [44]. This finding suggests that participation in school meals may be protective with regards to weight status for the most food insecure populations. It is possible that the protective effect of NSLP and SBP is not seen in more food secure populations due to increased access to foods outside of the school meals programs. The food insecure non-participants could be substituting the healthy foods/beverages offered as part of the NSLP and SBP with inexpensive, unhealthy foods or beverages (e.g., purchasing breakfast en route to school at corner store, leaving school at lunchtime for purchase of lunch at fast food restaurants). Also worth noting, because participation in school meals programs is not random, it is possible that the participants *vs.* non-participants are children from families in which health is prioritized and, thus, their overall dietary and other health behaviors are optimal, not just during school hours in which they access the quality foods provided by the SBP and NSLP. However, that conclusion cannot be made from these studies and should be investigated further. Second, children who ate breakfast in the classroom had significantly higher BMI-percentiles compared to children who ate breakfast in the cafeteria [39]. Reasons for this finding are unknown. It could be that because the classroom setting is more confined and structured children feel the need to eat the breakfast offered to them, even if they have already consumed breakfast. The potential environmental effects of this finding should be examined in future research.

Significant changes were made to the nutritional standards for the NSLP and SBP in 2010 [16]. Within the nutrition community there was concern that the more stringent nutritional standards would result in less appealing foods and thus a drop in participation in the school meals programs. Very few studies have been conducted since the most recent updates to the nutritional standards for the NSLP and SBP to assess nutritional content of foods, dietary intake of participants, and nutritional outcomes. One study assessed parental perceptions of the food and the associated odds of children participating in the programs and demonstrated that the odds of children consuming NSLP lunches were significantly lower if their parents perceived the meals to be somewhat unhealthy or very unhealthy [46]. Another study assessed potential changes in food selection, consumption, and waste after the new standards were implemented and showed that there was a significant increase in fruit selection, entrée consumption, and vegetable consumption and a significant decrease in milk selection [47]. The decrease in milk consumption seen may have been attributed to a district milk policy change and not the changes to the SBP and NSLP nutritional standards [47]. Further research should be conducted to determine if milk consumption is decreasing among SBP and NSLP participants due to the 2010 changes, which limited milk served to flavored or unflavored fat-free milk or unflavored low-fat milk. Finally, the most recent SNDA IV report shows that while the majority of surveyed schools met the standards for SBP breakfasts, only few met all of the nutrition standards for NSLP lunches [55], providing additional evidence in support of the need to develop strategies that would enable development of menus that meet the current standards.

Results from the current study demonstrate that the nutritional requirements for SFSP meals have not progressed equivocally. There are multiple notable differences in the school verses summer meal requirements for the CMPS that could significantly impact the nutritional quality of the meals served and consumed by the children. To this end, unlike the summer meal requirements, the school meals requirements include separate fruit and vegetable specifications, daily vegetable requirements, specification of a variety of fruits and vegetables, age-specific requirements, whole grain requirement, and fat, saturated fat, and sodium restrictions. The current SFSP requirements most similarly reflect the original meal pattern-based SBP and NSLP requirements demonstrating the critical need for policy change that would enable updates to the SFSP nutrition standards to match the requirements of the NSLP and SBP.

Due to the discovery that there are severely limited and outdated data on the nutritional quality of meals served as part of the SFSP, we conducted a case study on a sample SFSP menu from summer 2015 that included both breakfast and lunches. The nutritional analysis of this one-week menu demonstrated that SFSP meals met current SBP and NSLP standards for certain nutrients (e.g., fat and saturated fat for breakfast, calcium for both breakfast and lunch for all grade groups, and vitamin A for both breakfast and lunch for all group grade groups), and failed to meet the standards for others. The most concerning results (*i.e.*, deficiencies were noted for both breakfast and lunch and across all grade group categories) related to protein and carbohydrate (in excess) and fiber (insufficient), which if consumed at these levels could lead to weight gain, which is particularly troubling given the current trends of excess weight gain during the summer months among disadvantaged children living in the US [56,57,58,59,60]. That said, developing a menu that effectively meets the differing nutrient requirements of each grade group presents a significant challenge, highlighting the need to identify strategies that would overcome this problem.

Also of concern, results from the SFSP menu case study demonstrated that children were offered only two servings of vegetables over the course of the entire week. The situation is compounded if children are discarding vegetables as the 2003 USDA report indicates [45]. Given the critical role of vegetable intake in preventing chronic illness and low intake of vegetable among US. children and adolescents—half to one third of the recommended number of daily servings according to the 2011–2012 What We Eat in America survey—these data are particularly alarming [61,62].

There are several limitations to this review. First, nearly all of the studies were observational and cross-sectional in design, with the exception of two studies that took a prospective approach. As such, cause and effect relationships cannot be determined and there is the potential of reverse causality of the findings demonstrated. Unfortunately, this will likely continue to be the reality of research efforts in this particular subject area since the NSLP, SBP, and SFSP are national, public health programs that were established almost 75 years ago and it is not feasible to retrospectively assess data from non-participants that became participants. Additionally, it would be unethical to conduct a study that required participants to deny the benefits of these programs.

Other limitations to the data utilized in the studies included in this review are potential biases. As previously noted, there is a high potential for reporting bias. A significant amount of the data utilized in these studies was derived from large, national data sets that consist of predominantly self-reported data, which may lack trustworthiness [63], though others provide evidence in favor of their validity for certain nutrients [64]. A second form of inevitable bias in this line of work is participant bias. There is no ethical or feasible way to randomize children to receive school meals. Thus, school meals recipients may have other underlying characteristics that could be affecting or causing the outcomes of interest.

## 5. Conclusions

This was the first study of its kind to review the complete evidence surrounding the nutritional quality of USDA CMP meals and the potential nutritional effects of participation in CMPs. In addition, this study provides a unique context by presenting the data in direct relation to the historical progress of the SBP, NSLP, and SFSP nutritional standards. Taking a historical perspective on the research allows for the opportunity to determine if in fact the USDA CMPs are meeting their goal of improving the nutritional health of the current generation of US children, providing information that could be used: (a) for the basis of potential policy updates; as well as (b) to direct the “next steps” for future research in this area of inquiry. Even though the research studies under review in the current study were not specifically designed to assess the impact of changes to the programs on (a) nutritional adequacy of program meals; (b) dietary intakes of participants; and (c) nutritional outcomes of participants, examining the research along a time continuum in correspondence with the changes in nutrition standards to the programs allows for the opportunity to observationally examine if the changes in standards over time have led to improvements in the diet and health of its participants, which could serve as preliminary for a larger, more rigorously designed study.

Several unique findings from this review should be investigated in follow up studies, such as the potential protective effect of SBP participation, the environmental effect of where breakfast is consumed, and differences in food security and potential protective effects of school meals participation on weight status. Researchers should also consider utilizing biomarkers to provide more concrete evidence as to the nutritional status of SBP, NSLP, and SFSP participants compared to non-participants, while assuring similarity between groups in demographic, health, and nutritional characteristics at baseline.

In addition, more research needs to be conducted in several key areas. First, meal quality, dietary intake, and nutritional outcomes should be investigated as a result of the more stringent nutritional requirements implemented in 2010. Secondly, due to the complete dearth of research conducted on the SFSP, investigators should explore the nutritional quality and variety of foods served during the SFSP, as well as the impacts on nutritional outcomes such as dietary intake, weight status, and other anthropometrics as a potential avenue for policy change to improve the nutritional state of our country’s children today.
